# *NSD1* Mutations in Sotos Syndrome Induce Differential Expression of Long Noncoding RNAs, miR646 and Genes Controlling the G2/M Checkpoint

**DOI:** 10.3390/life12070988

**Published:** 2022-07-02

**Authors:** Giuseppina Conteduca, Davide Cangelosi, Simona Coco, Michela Malacarne, Chiara Baldo, Alessia Arado, Rute Pinto, Barbara Testa, Domenico A. Coviello

**Affiliations:** 1 Laboratory of Human Genetics, IRCCS Istituto Giannina Gaslini, 16147 Genoa, Italy; giusy_conteduca@alice.it (G.C.); michelamalacarne@gaslini.org (M.M.); chiarabaldo@gaslini.org (C.B.); alessiaarado@gaslini.org (A.A.); ruteluisapinto@gmail.com (R.P.); testabarbara@libero.it (B.T.); 2 Clinical Bioinformatics Unit, IRCCS Istituto Giannina Gaslini, 16147 Genoa, Italy; davidecangelosi@gaslini.org; 3 Lung Cancer Unit, IRCCS Ospedale Policlinico San Martino, 16132 Genoa, Italy; simona.coco@hsanmartino.it

**Keywords:** G2/M checkpoint, Sotos syndrome, gene expression, *NSD1*, noncoding RNAs

## Abstract

An increasing amount of evidence indicates the critical role of the *NSD1* gene in Sotos syndrome (SoS), a rare genetic disease, and in tumors. Molecular mechanisms affected by *NSD1* mutations are largely uncharacterized. In order to assess the impact of *NSD1* haploinsufficiency in the pathogenesis of SoS, we analyzed the gene expression profile of fibroblasts isolated from the skin samples of 15 SoS patients and of 5 healthy parents. We identified seven differentially expressed genes and five differentially expressed noncoding RNAs. The most upregulated mRNA was stratifin (*SFN*) (fold change, 3.9, Benjamini–Hochberg corrected *p* < 0.05), and the most downregulated mRNA was goosecoid homeobox (*GSC*) (fold change, 3.9, Benjamini–Hochberg corrected *p* < 0.05). The most upregulated lncRNA was lnc-C2orf84-1 (fold change, 4.28, Benjamini–Hochberg corrected *p* < 0.001), and the most downregulated lncRNA was Inc-C15orf57 (fold change, −0.7, Benjamini–Hochberg corrected *p* < 0.05). A gene set enrichment analysis reported the enrichment of genes involved in the KRAS and E2F signaling pathways, splicing regulation and cell cycle G2/M checkpoints. Our results suggest that *NSD1* is involved in cell cycle regulation and that its mutation can induce the down-expression of genes involved in tumoral and neoplastic differentiation. The results contribute to defining the role of *NSD1* in fibroblasts for the prevention, diagnosis and control of SoS.

## 1. Introduction

The nuclear receptor SET domain protein 1 (*NSD1*) histone methyltransferase has been identified as a protein that interacts with several nuclear receptors, and it may also act as a bifunctional transcriptional cofactor, playing a dual role in transcription [1,2]. The mono- and di-methylation of histone 3 lysine 36 (H3K36) and lysine 168 (H3K168) have been proposed to be the major cellular NSD1 substrates [3,4]. In addition, *NSD1* (NM 022455.4) can act as a tumor suppressor gene [5,6]. The structure of the NSD1 protein is characterized by two nuclear interaction domains (NIDs); two proline–tryptophan–tryptophan–proline (PWWP) domains; five plant homeodomains (PHD); an atypical plant homeodomain (C5HCH) finger; and a catalytic domain (CD), which is composed of pre-SET, subset of SET and post-SET-containing proteins. A so-called ‘associated with SET’ (AWS) domain is sometimes found instead of the pre-SET domain, containing Su(var)3–9, enhancer-of-zeste, Trithorax (SET) and a post-SET domain [7].

Germline mutations in the *NSD1* gene cause Sotos syndrome (SoS) (OMIM 117550), a rare genetic disease with a prevalence of 1/14.000 births. It is characterized by overgrowth, macrocephaly, advanced bone age, characteristic facial features (long face) and intellectual delay [8,9,10]. SoS is due to an *NSD1* gene haploinsufficiency that can be caused by several different mechanisms (truncating mutations, missense mutations, splice-site mutations, partial gene deletions and 5q35 microdeletions) [11,12]. Intragenic mutations account for 80–85% of SoS cases among European and American populations; 5q35 microdeletions are present in 10–15% of European and American cases and in more than 50% of Japanese cases [11].

In SoS patients bearing germline *NSD1* haploinsufficiency, a deregulation of the mitogen-activated protein kinase (MAPK)/ERK signaling pathway has been observed downstream of KRAS activation, and this has been postulated to contribute to accelerated skeletal outgrowth [13]. Even if different functions of NSD1 have been documented, the molecular mechanisms that induce the phenotypic characteristics of SoS remain largely unknown.

The Human Genome Project showed that approximately 76% of the human genome is transcribed, with about 70% of transcripts corresponding to noncoding RNAs [14,15]. Noncoding RNAs can be divided into two groups depending on their lengths. Small RNAs are defined as being shorter than 200 nucleotides (nt), which include microRNA, and RNAs longer than 200 nt are referred to as long noncoding RNAs (lncRNAs). lncRNAs and microRNAs have roles in different mechanisms of gene regulation [16,17,18,19,20,21,22,23].

Moreover, somatically acquired alterations are frequently detected in various human cancers [24,25,26,27,28,29,30,31,32,33,34], and RNAi-mediated knock-down seems to increase the proliferation of tumor cells, suggesting the role of *NSD1* as a tumor suppressor [5]. Studies have also described NSD1 as a potential biomarker for drug resistance in tumors.

In this study, we generated genome-wide transcription profiles for fibroblasts taken from SoS patients and their sex-matched healthy parents in order to identify which lncRNAs, microRNAs, mRNAs and downstream pathways are perturbed by NSD1 mutations in SoS syndrome. Our results underline the involvement of NSD1 in the G2/M cell cycle checkpoint, which prevents cells from entering mitosis when DNA is damaged, stopping cell proliferation. We also observed that NSD1 mutations can induce dysregulation in the expression of noncoding RNA.

## 2. Materials and Methods

### 2.1. Patients

This study was conducted with the approval of the Ethical Committee of the Liguria Region (Approval #OG01IGG, 12 July 2021). Written informed consent was obtained from all participants. Fifteen individuals with the classic clinical features of SoS and with molecular diagnoses of *NSD1* pathogenic variants (point mutation or deletion) were enrolled in this study. As controls, five parents with the same sexes as their co-respective SoS patients, selected on the basis of skin biopsy availability, were also enrolled. The details are presented in Tables S1 and S2. The fibroblast cell lines were deposited at the Gaslini Biobank. 

### 2.2. Cell Culture 

Skin fibroblasts were obtained via punch biopsy of the forearm skin. Cells were maintained in the RPMI-1640 medium (Life Technologies, Grand Island, NY, USA), supplemented with 10% fetal calf serum (FCS), 2 mM L-glutamine, 100 U/mL penicillin and 100 μg/mL streptomycin (Euroclone S.p.a, Milan, Italy). Cell lines were tested for mycoplasma with a mycoplasma detection kit (Lonza, Basel, CH, Switzerland). In all experiments, cells with between 2 and 15 passages were used. 

### 2.3. Analysis of NSD1 Pathogenetic Variants 

DNA was extracted from the fibroblasts using QIAamp^®^ DNA Blood kit (Qiagen, Milan, Italy) according to the manufacturer’s protocol. PCR amplifications were performed using platinum-Taq DNA polymerase (Thermo Fisher Scientific, Carlsbad, CA, USA) and specific primers for the 23 different NSD1 exons (see Table S3). The PCR conditions were a single denaturation step at 95 °C for 3 min followed by 40 cycles of 95 °C/30 s, 58 °C/30 s and 72 °C (1 min/kb), with a final extension step at 72 °C for 5 min. PCR products were sequenced using the ABI BigDye Terminator Ready Reaction Mix (Thermo Fisher Scientific, Foster City, CA, USA) and analyzed on an ABI 3130XL Genetic Analyzer (Applied Biosystems, Foster City, CA, USA) according to the manufacturer’s instructions. The sequences were aligned with Seqscape analysis software V.2.5 (Thermo Fisher Scientific, Foster City, CA, USA). To verify variants with deletions of the 5q35.3 region, array-CGH was performed with CGH 8 × 60 K (Agilent Technologies, Santa Clara, CA, USA) according to the manufacturer’s protocol. The data were analyzed with the Agilent Cytogenomics 4.0.3.12 software (Agilent Technologies, Santa Clara, CA, USA). All genomic positions were reported according to the human genome assembly (GRCh37/hg19).

### 2.4. Gene Expression Profiling

The total RNA was extracted using Trizol reagent (Thermo Fisher Scientific, MA, USA). The total RNA from each sample was quantified with the NanoDrop ND-1000, and RNA integrity was assessed by an Agilent 2100 bioanalyzer. Microarray hybridization was performed using the Agilent One Color microarray gene expression kit and the SurePrint G3, 8 × 60 K Human Gene Expression V3 array (Agilent Technologies, Santa Clara, CA, USA) following established protocols. Briefly, cyanine-3-CTP-labeled cRNA was hybridized onto SurePrint G3 microarray chips (Design ID: G4851C), which contained 50,599 probes for 32,776 human mRNAs and 17,438 human lncRNAs; these were derived from authoritative databases, including RefSeq, Ensemble, GenBank and the Broad Institute. After hybridization and washing, processed slides were scanned with a Agilent microarray scanner (Agilent Technologies). Raw data were extracted using Feature Extraction (version 12.0.1.1; Agilent Technologies). Next, data preprocessing, including normalization and filtering, was carried out with the Genespring software (version 14.3; Agilent Technologies). Raw data were normalized by a 75-percentile shift, log2-transformed and shifted to the median of all samples. The microarray data were deposited in GEO with the accession number GSE204775 (http://www.ncbi.nlm.nih.gov/geo/query/acc.cgi?acc=GSE204775, accessed on 24 May 2022). Only samples in which quality control was excellent were used for subsequent analyses in order to reduce potential biases introduced by analyzing low-quality specimens. 

### 2.5. Quantitative Real-Time RT-PCR Validation of Microarray Gene Expression Patterns

To confirm the significant modulation of the differentially expressed genes resulting from microarray data analysis, we performed real-time quantitative PCR using gene-specific primer (Table S3) and ran each experiment in triplicate for analysis robustness. Briefly, cDNA was synthesized from 400 ng of the total RNA using the Advantage RT cDNA Kit (Clontech, Mountain View, CA, USA) following the manufacturer’s instructions. Specifically, samples were incubated at 42 °C for 90 min, followed by 2 min at 90 °C. Quantitative real-time PCR was performed using LightCycler 480 SYBR Green I Master (Roche Diagnostics, Mannheim, Germany) in a 15 µL reaction mixture. β-actin was used as an endogenous reference to normalize gene expression values with the 2^−ΔΔCt^ method [35]. PCR products were also confirmed via sequence analysis using the ABI BigDye Terminator Ready Reaction Mix (Life Technologies, Carlsbad, CA, USA) and analyzed on an ABI 3130XL genetic analyzer (Life Technologies, Carlsbad, CA, USA) according to the manufacturer’s protocol. 

### 2.6. Bioinformatic Analysis

Using a bioinformatic analysis, based on the normalized fluorescence signal values of the lncRNA/mRNA probes, differentially expressed mRNAs and lncRNAs were identified by examining fold changes as well as by *p*-values calculated via Student’s t-tests between the SoS patients and the healthy controls using the “Statistical Analysis” module from the analysis section, provided by the Genespring software (version 14.3; Agilent Technologies) with default parameters. The PO value was adjusted with the Benjamini–Hochberg method to account for the increased probability of false positive findings derived from multiple comparisons. A fold change of ≥2.0 or ≤0.5 and a p-adjusted value of <0.05 were considered to be significantly up- or downregulated. In silico protein–protein functional interactions among the differentially expressed genes were assessed with the STRING database (http://stringdb.org, accessed on 24 May 2022) [36] using default parameters. An extension of the network was analyzed to assess potential indirect interactions between the differentially expressed genes. Using and Graphpad Prism 8 software, a Bonferroni-adjusted significance level of 0.05 was calculated to account for the increased possibility of obtaining false positive results.

A gene set enrichment analysis (GSEA) [37] was used to assess the enrichment of functionally related gene sets in SoS samples [37]. Chemical and genetic perturbations (C2.CGP), hallmark (H), gene ontology biological processes (C5.GO.BP) or gene set collections retrieved from the Molecular Signature Database (MSigDB) v7.4 [38] were used to perform enrichment analyses. GSEA calculated an enrichment score (ES) and a normalized enrichment score (NES) for each gene set and estimated the statistical significance of the NES with an empirical permutation test using 1000 gene permutations in order to obtain the nominal *p*-value (NOM *p*-value). When multiple gene sets are evaluated, GSEA adjusts the estimate of the significance level to account for multiple hypothesis testing. To this end, GSEA computed the false discovery rate *q*-value (FDR *q*-value), measuring the estimated probability that the normalized enrichment score represented a false positive finding. We considered gene sets containing between 15 and 250 genes. Gene sets with nominal *p*-values lower than 0.05 and FDR *q*-values lower than 0.05 were considered significantly enriched. 

## 3. Results

### 3.1. Comparison of the Gene Expression Profile of Fibroblast Lines between SoS and Control Individuals

**Figure 1:**
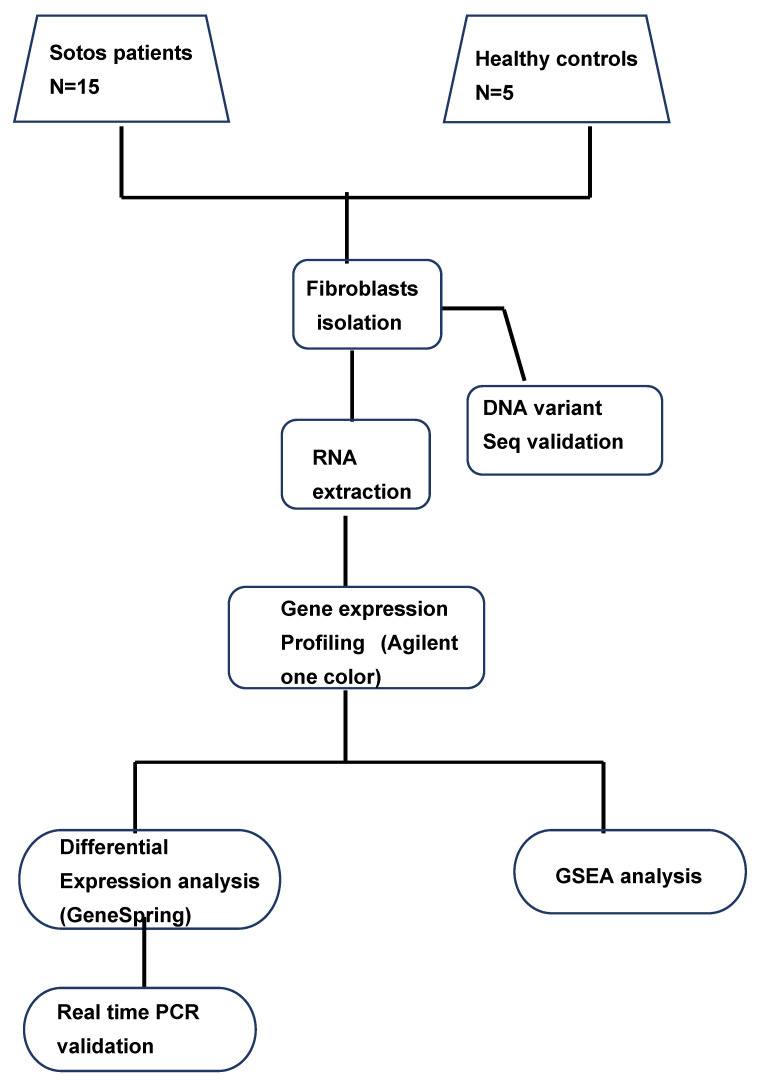
Schematic representation of the whole study strategy used. A GeneSpring differential expression analysis assessed the significant modulation of target genes between the SoS patient group and the healthy control. Differentially expressed genes were validated by real-time PCR. A GSEA pathway analysis identified the most significantly altered biological processes and pathways.

Fifteen patients with SoS and five healthy parents were enrolled in the study. The characteristics of the enrolled individuals are reported in Tables S1 and S2. A flowchart summarizing the main steps of these analyses is shown in Figure 1. RNA obtained from dermal fibroblasts from those SoS patients with confirmed NSD1 alterations and their sex-matched controls were analyzed using Agilent SurePrint G3 microarrays. Gene expression profiles were collected into one dataset for subsequent analysis. 

Quality control was carried out on all samples to exclude potential artifacts derived from sample preparation and processing. One sample from the SoS patient group was filtered out because it did not pass the conformity test (Supplementary Figure S1). A differential expression analysis identified five significantly differentially expressed noncoding RNAs (four lncRNA and one microRNA) (Table 1) and seven probe sets relative to the mRNAs (Table 2). Four probe sets relative to the noncoding RNAs were upregulated (*MIR646*, *lnc-C2orf84-1*, *lnc-C00665* and *lnc-C20orf197-3*) and one was downregulated (*lnc-C15orf57*, fold change, −0.7, *p* < 0.05). The most upregulated lncRNA was associated with *lnc-C2orf84-1* (fold change, 4.28, *p* < 0.001; Table 1). Among the probe sets relative to mRNAs, four were upregulated (*NOS3, CD19, SFN* and *ZNF883*) and three were downregulated (*NDRG2, GSC* and *SORBS1*). The most upregulated probe set was associated with the stratifin (*SFN*) gene (fold change, 3.9, *p* < 0.05), and the most downregulated was associated with the goosecoid homeobox (*GSC*) gene (fold change, 3.9, *p* < 0.05; Table 1). 

**Figure 2:**
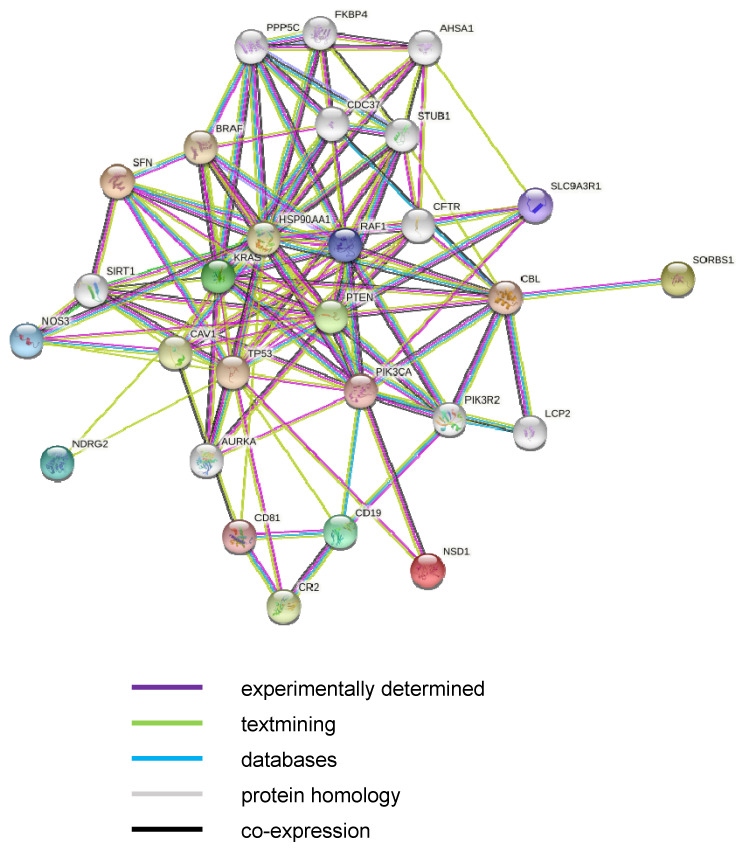
Functional interaction network among differentially expressed genes between SoS patients and healthy controls.

To assess the biological connection between the differentially expressed genes, we performed a network analysis using the STRING-DB software [36]. We extended the network to assess likely indirect interactions between differentially expressed genes. The resulting network, reported in Figure 2, showed that, statistically, the significantly modulated genes did not display any direct interaction with NSD1, but it did report an interaction with a subset of other gene products, including KRAS and pTEN, which are known to interact with NSD1. These findings indicate that NSD1 is an important regulator of different genes involved in cell differentiation and proliferation. 

### 3.2. Validation of Microarray Results by Real-Time Quantitative PCR

**Figure 3:**
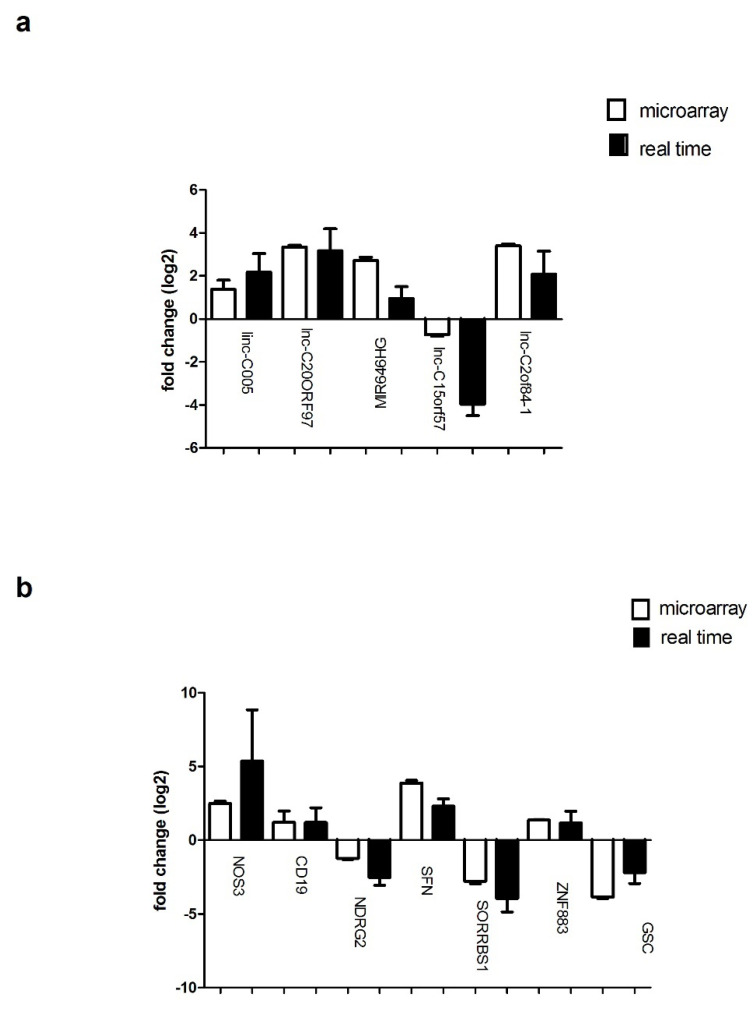

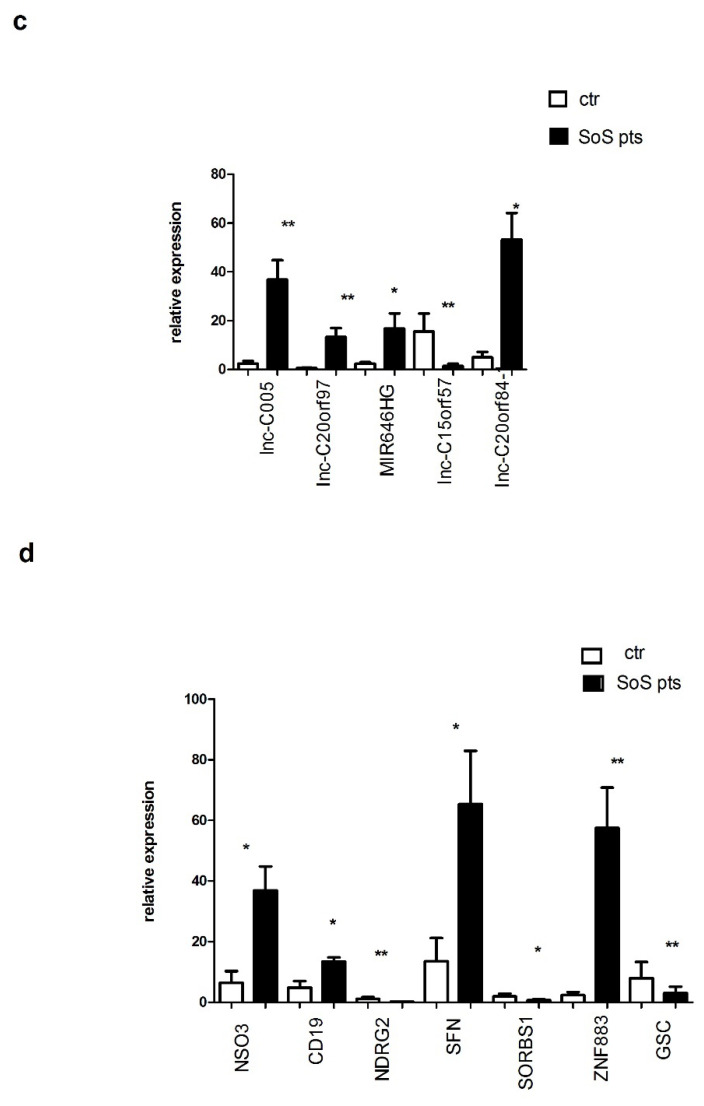
Real-time PCR validation of differentially expressed genes. (**a**) Quantitative expression measurements of five noncoding RNAs (i.e., *lnc-C15orf57*, *MIR646*, *lnc-C2orf84-1*, *Inc-C00665* and *lnc-C20orf197-3*) and (**b**) seven mRNAs (i.e., *NOS3*, *ZNF883*, *CD19*, *SFN*, *GSC*, *NDRG2* and *SORBS1*). The fold change represents the average difference in expression level of the respective genes between the SoS fibroblasts and the controls. The black bars depict the fold changes detected with the microarray, whereas the white bars show the average fold change with respect to the triplicate real-time PCR assessments. The fold changes indicating a downregulated expression are represented with negative values. The error bars represent the standard error of the mean. Significant levels were at *p* < 0.05. (**c**) Compared to healthy controls, five noncoding RNAs (*lin-C005, Inc-C20orf97*, *MIR646HG*, *lnc-C15orf57* and *Inc-C20orf84-1*) and (**d**) seven mRNAs (*NOS3*, *ZNF883*, *CD19*, *SFN*, *NDRG2*, *SORBS1* and *GSC*) were selected. The black bars depict healthy control (Ctr), and the white bars represent the Sotos syndrome patients (SoS pts). The results were consistent with the findings obtained from the microarray analysis. Data are presented as the mean ± standard deviation. * *p* < 0.05, ** *p* < 0.01.

All seven genes and six lncRNAs that were found to be significantly modulated in the microarray analysis were subjected to further validation using real-time PCR. Interestingly, the qPCR results confirmed all of the deregulations in the genes and lncRNAs assessed by microarray analysis (Figure 3a,b). In particular, a Student’s *t*-test analysis, by applying a 0.05 cutoff on the Benjamini–Hochberg-corrected *p*-values, showed that the expressions of *MIR646* (*p* < 0.05), *lnc-C2orf84-1* (*p* < 0.05), *lnc-C00665* (*p* < 0.001), *lnc-C20orf197-3* (*p* < 0.001), *NOS3* (*p* < 0.05), *ZNF883* (*p* < 0.001), *CD19* (*p* < 0.05) and *SFN* (*p* < 0.05) were upregulated and that those of *GSC* (*p* < 0.05), *lnc-C15orf57* (*p* < 0.001), *NDRG2* (*p* < 0.001) and *SORBS1* (*p* < 0.05) were downregulated in SoS patients.

### 3.3. Gene Set Enrichment Analysis

Gene set enrichment analysis (GSEA) is a computational technique that assesses the coordinated gene expression modulation of functionally related genes (gene sets) between two groups [37]. To perform this analysis, we used gene sets included in the chemical and genetic perturbation, hallmark (H) and C2 gene ontology collections from the MSigDB database [38]. The analysis showed the 89 significantly enriched biological processes and pathways in the NSD1-mutated fibroblasts of SoS patients, as compared to the healthy controls; Table S4 reports the complete list of significant processes and pathways. The most statistically significant over-represented gene sets were related to the cell cycle and proliferation, cell differentiation, P53-mediated cell cycle arrest, cellular senescence and cancer (Table 3). In particular, upregulated genes were mainly involved in the regulation of the meiotic cell cycle, the negative regulation of nuclear division (Supplementary Figure S2A,B) and kinetochore organization. The most statistically significant underrepresented gene sets were related to the E2F pathway (Supplementary Figure S2), pediatric cancer markers, neoplastic transformation via the STAT3 pathway, MYC and TFRC targets, the cell cycle G2/M checkpoint, TNFA-signaling via NFKB, epithelial mesenchymal transition, the apoptosis process caused by CDKN1A via TP53 and neoplastic transformation KRAS signaling pathways (Supplementary Figure S3A,B). 

Then, an interaction network was generated using the STRING database. Nodes represent gene products, and edges represent protein–protein associations. Only the associations with an evidence score higher than 0.3 are shown; their colors indicate different kinds of interaction evidence (key, bottom right). 

## 4. Discussion

SoS is an autosomal dominant disorder with manifestations characterized by tall stature, facial dysmorphism and mental retardation [1,2,3,4,5]. 

Currently, the pathogenetic cause of SoS is thought to be due to haploinsufficiency in the NSD1 gene [6]. However, the molecular mechanisms of action are still unknown. At present, an increasing number of studies have indicated that NSD1 is involved in the regulation of a wide variety of biological processes [27,28,29,30,31,32]. The *NSD1* gene is expressed in most tissues from different organisms. Elevated NSD1 levels are detected in normal brains, pancreases and male reproductive tracts, in hematopoietic organs, bone marrow and lymphoid tissues [9]. Significant *NSD1* mRNA expression in bone marrow polymorphonuclear cells, CD4, CD8 and NK has also been reported by genevisible.com [10].

*NSD1* defects induce the alternate methylation of H3K36, blocking cellular differentiation and promoting oncogenesis [24]. Reduced *NSD1* activity has been observed in head and neck cell cancers, while abnormal DNA-promoter hypermethylation is associated with renal clear-cell carcinoma [25,27,28,29,30]. Furthermore, *NSD1* mutations are present in hematological malignancies, and a recurrent t(5;11)(q35;p15) chromosomal translocation is associated with aggressive pediatric acute myeloid leukemia (AML). The resulting fusion protein with leukemogenic activity is composed of the N-terminus of nucleopore 98 (NUP98) and the C-terminal NSD1 SET domain [32,33,34]. The abnormal expression of *NSD1* in different cancers has also been detected, which is associated with tumorigenesis, survival and chemoresistance. In pancreatic ductal adenocarcinoma, a high level of NSD1 is significantly correlated with the advanced clinical stage [39]. Moreover, *NSD1* gene-silencing inhibits the proliferative, migratory and invasive abilities of hepatocellular carcinoma cells [40]. Furthermore, upregulation of NSD1 might improve oncogenic initiation through the reinforced methylation of H3K36 [41].

In the present study, the expression of lncRNAs and mRNAs in SoS patients and the healthy control was investigated using a microarray analysis to reveal their potential role in the pathogenesis of SoS. We decided to compare the expression profiles of SoS patients with those of their healthy parents, as they share approximately 50% of their DNA; this allowed us to minimize differences caused by other genes variants with respect to gene expression [42,43].

In total, five noncoding RNAs and seven mRNAs were identified as being differentially expressed. GSEA analyses were used to explore the possible biological functions and potential mechanisms of the mRNAs and noncoding RNAs in SoS.

The results of the present study demonstrated that these biological processes, including G2/M checkpoints, can control transitions between cell cycle phases, cell senescence, and meiotic and mitotic division and are among the most significantly enriched mRNAs in SoS. Most of these functions are involved in neoplastic disease and tumor development, and this is consistent with previous studies [24,25,26,27,28,29,30,31,32,33,34,39,40,41]. Furthermore, we observed that the KRAS signaling pathway, the E2F target and apoptosis by CDKN1A via TP53-signaling were remarkably downregulated in the SoS samples compared to the healthy controls. On the other hand, genes involved in the regulation of nuclear division, meiotic cell cycle and kinetochore organization were remarkably upregulated in SoS patients. 

lncRNAs have long been considered the only transcriptional noise; however, several lncRNAs analyzed with microarrays showed greater expression in immortalized lymphocytes [44]. lncRNAs were also found to be upregulated in neuroblastoma cell line LAN-I [45,46]. Different studies have shown that lncRNA alterations contribute to neuronal and neurodegenerative disease development [19,47,48]. lncRNAs may provide basic information that could be used to understand pathways related to the disease course of SoS and to find more effective targeted therapies.

The results of the present study revealed that, among the differentially expressed genes, SFN was the most upregulated mRNA (FC = 3.9) in SoS samples. The SFN protein is ubiquitously expressed and exerts many relevant intracellular functions, such as the control of cell cycle and apoptosis, the regulation of signal transduction pathways, cellular trafficking, cell proliferation and differentiation, and protein folding and processing, among others [49]. Both p53 and SFN prevent DNA errors during mitosis [40,41,42,43,44,45,46,47,48,49,50,51,52]. Therefore, according to the reported evidence, our study suggests that NSD1 may be an important protein for cell cycle and mitotic translation, regulating *SFN* G2/M checkpoint expression.

In addition, in our study, *GSC*, *SORBS1* and *NDRG2* mRNA were down-expressed in Sotos patients compared to the healthy controls. These three genes encode proteins that are associated with the actin cytoskeleton, the formation of actin stress fibers and focal adhesions, and receptor tyrosinase kinase-signaling, and they control different aspects of morphogenesis and cell differentiation [53,54,55]. Some studies have reported that the expression of *GSC* inhibits the proliferation of PC12 cells and that—as with *NSD1—GSC* mediates the regulation of erythropoiesis functions [56]. *NDRG2* is frequently downregulated in cancer, and it plays an important role in the control of tumor growth and metastasis [57]. SORBS1 expression has been detected in colorectal cancer cell lines, and its overexpression increases the proliferation and migration abilities of tumor cells [58].

We found a downregulation of the *SORBS1*, *NDRG2* and *GSC* genes in the SoS samples, similar to the RNA expression profile of the mesenchymal cell. The down-expression of these three genes induces the epithelial–mesenchymal transition [59,60].

Furthermore, *GSC* acts in mesenchyme-derived tissues during craniofacial development, and it has been reported that mice that are homozygous for the deletion of the homeobox gene goosecoid can have multiple craniofacial defects, and various bone and cartilage malformations [61,62]. Therefore, we propose that GSC down-expression may contribute to the advanced bone age and characteristic facial features of Sotos syndrome, including macrocephaly and long face.

Epilepsy is one of the clinical manifestations found in SoS patients. We found an over-expression of ZNF833, a gene that is correlated with the progression of epilepsy in rats [63].

In conclusion, we propose that NSD1 may participate in Sotos syndrome’s mechanism of action by regulating the function of lncRNA, inducing the down-expression of GSC, NDRG2 and SORBS1 and the up-expression of SFN and ZNF883.

These expression signatures may be useful tools for screening and monitoring the disease and for predicting its prognosis.

Additional investigations should be performed to establish and clarify the detailed molecular mechanism of action behind the lncRNA-mediated regulation of potential coding genes in SoS. Further studies with a larger sample size should also be performed to verify the relevant results.

## Figures and Tables

**Table 1:** Differentially expressed probe sets relative to noncoding RNAs (ncRNAs) in SoS samples.

Nr	Probe Set ID	Gene Symbol	Gene Name	Seq. Name	Log_2_ Fold Change	*p*-Value	*p*-Value Adjusted
1	A_22_P00002837	Lnc-C20orf197-3	long intergenic nonprotein-coding RNA	lnc-C20orf197-3:13	3.2	4.95 × 10^−7^	0.007
2	A_22_P00002837	Lnc-C2orf84-1	long intergenic nonprotein	lnc-C2orf84-1:1	3.2	4.95 × 10^−7^	0.007
3	A_22_P00002715	MIR646HG	MIR646 host gene	ENSG00000228340	2.8	8.64 × 10^−6^	0.042
4	A_24_P16214	LINC00665	long intergenic nonprotein-coding RNA 665	ENST00000427868	1.2	2.57 × 10^−6^	0.016
5	A_32_P98975	C15orf57	chromosome 15 open reading frame 57	NM_052849	−0.7	1.40 × 10^−5^	0.047

**Table 2:** Significant differentially expressed probe sets relative to mRNAs in SoS samples.

Nr	Probe Set ID	Gene Symbol	Gene Name	Seq. Name	Log2 Fold Change	*p*-Value	*p*-Value Adjusted
1	A_33_P3389286	*SFN*	stratifin	NM_006142	3.99	1.62 × 10^−6^	0.015
2	A_23_P113572	*CD19*	CD19 molecule	NM_001770	3.36	1.28 × 10^−6^	0.014
3	A_33_P3305790	*NOS3*	nitric oxide synthase 3 (endothelial cell)	NM_000603	2.4	1.41 × 10^−7^	0.0027
4	A_22_P00017915	*ZNF883*	zinc finger protein 883	NM_001101338	1.33	4.95 × 10^−6^	0.026
5	A_33_P3334515	*NDRG2*	NDRG family member 2	NM_001282213	−1.24	1.06 × 10^−5^	0.047
6	A_24_P317907	*SORBS1*	sorbin and SH3 domain containing 1	NM_001034954	−3.1	1.14 × 10^−5^	0.047
7	A_23_P76774	*GSC*	goosecoid homeobox	NM_173849	−3.98	1.38 × 10^−5^	0.047

**Table 3:** Selected significantly enriched gene sets in SoS samples found via GSEA.

Pathway or Process Description ^a^	Number of Genes ^b^	FDR *q*-Value ^c^	Type of Regulation ^d^	Ontology ^e^
**Cancer Process**				
**Florio neocortex basal radial glia DN**	151	0.00	DOWN	CGP
**Kong E2F3 targets**	77	0.00	DOWN	CGP
**Kobayashi EGFR-signaling 24 h DN**	213	0.00	DOWN	CGP
**Whitfield cell cycle literature**	39	0.00	DOWN	CGP
**Whiteford pediatric cancer markers**	102	0.00	DOWN	CGP
**Nakayama soft-tissue tumors PCA2 UP**	76	0.00	DOWN	CGP
**Zhan multiple myeloma pr UP**	35	0.00	DOWN	CGP
**Pyeon HPV positive tumors UP**	79	0.00	DOWN	CGP
**Sotiriou breast cancer grade 1 vs. 3 UP**	127	3.65 × 10^−4^	DOWN	CGP
**Chiang liver cancer subclass proliferation UP**	146	8.15 × 10^−4^	DOWN	CGP
**Farmer breast cancer cluster 2**	29	0.001	DOWN	CGP
**Villanueva liver cancer KRT19 UP**	147	0.002	DOWN	CGP
**Rodrigues thyroid carcinoma DN**	66	0.002	DOWN	CGP
**Finetti breast cancer kinome red**	15	0.002	DOWN	CGP
**Riggi Ewing sarcoma progenitor DN**	158	0.002	DOWN	CGP
**Azare neoplastic transformation by STAT3 DN**	16	0.002	DOWN	CGP
**West adrenocortical tumor markers UP**	103	0.005	DOWN	CGP
**Li Wilms tumor anaplastic UP**	15	0.010	DOWN	CGP
**Li prostate cancer epigenetic**	27	0.014	DOWN	CGP
**GAL leukemic stem cell DN**	188	0.014	DOWN	CGP
**Rickman head and neck cancer B**	37	0.018	DOWN	CGP
**Poola invasive breast cancer UP**	228	0.019	DOWN	CGP
**Sengupta nasopharyngeal carcinoma UP**	240	0.024	DOWN	CGP
**SMID breast cancer luminal A UP**	68	0.025	DOWN	CGP
**Vantveer breast cancer BRCA1 UP**	28	0.027	DOWN	CGP
**Winnepenninckx melanoma metastasis UP**	134	0.029	DOWN	CGP
**Ferreira Ewing’s sarcoma unstable vs. stable UP**	131	0.029	DOWN	CGP
**Lopes methylated in colon cancer UP**	22	0.047	DOWN	CGP
**Chiaradonna neoplastic transformation KRAS UP**	113	0.047	DOWN	CGP
**Cell Cycle and** **Proliferation Process**				
**Fischer G2 M cell cycle**	191	0.00	DOWN	CGP
**Croonquist NRAS signaling DN**	55	0.00	DOWN	CGP
**Molenaar targets of CCND1 and CDK4 DN**	48	0.00	DOWN	CGP
**Ishida E2F targets**	39	0.00	DOWN	CGP
**Reichert mitosis LIN9 targets**	21	0.00	DOWN	CGP
**Graham normal quiescent vs. normal dividing DN**	74	1.15 × 10^−4^	DOWN	CGP
**Odonnell TFRC targets DN**	110	2.18 × 10^−4^	DOWN	CGP
**Graham cml dividing vs. normal quiescent UP**	157	0.002	DOWN	CGP
**Eguchi cell cycle RB1 targets**	17	0.002	DOWN	CGP
**Yu MYC targets UP**	40	0.003	DOWN	CGP
**Zhou cell cycle genes in IR response 24HR**	103	0.003	DOWN	CGP
**Odonnell targets of MYC and TFRC DN**	36	0.006	DOWN	CGP
**Alcalay AML by NPM1 localization DN**	158	0.010	DOWN	CGP
**Zhou cell cycle genes in IR response 6HR**	70	0.011	DOWN	CGP
**Plasari TGFB1 targets 10HR DN**	205	0.014	DOWN	CGP
**Chicas RB1 targets growing**	196	0.02	DOWN	CGP
**ULE-splicing via NOVA2**	35	0.02	DOWN	CGP
**Benporath proliferation**	124	0.033	DOWN	CGP
**Whitfield cell cycle G2 M**	162	0.034	DOWN	CGP
**Liang silenced by methylation 2**	50	0.034	DOWN	CGP
**Nojima SFRP2 targets DN**	19	0.034	DOWN	CGP
**Kamminga EZH2 targets**	37	0.037	DOWN	CGP
**Graham CML quiescent_VS normal quiescent UP**	74	0.040	DOWN	CGP
**GOBP heat generation**	15	0.014	UP	GO BP
**GOBP regulation of meiotic cell cycle**	31	0.07	UP	GO BP
**GOBP negative regulation of nuclear division**	42	0.016	UP	GO BP
**GOBP regulation of feeding behavior**	20	0.021	UP	GO BP
**GOBP positive regulation of organic acid transport**	29	0.017	UP	GO BP
**GOBP regulation of nuclear division organization**	106	0.046	UP	GO BP
**GOBP kinetochore organization**	16	0.041	UP	GO BP
**HALLMARK G2M checkpoint**	172	0.001	DOWN	H
**HALLMARK KRAS-signaling UP**	174	0.0001	DOWN	H
**HALLMARK mitotic spindle**	163	0.008	DOWN	H
**HALLMARK inflammatory response**	159	0.006	DOWN	H
**HALLMARK E2F targets**	167	0.005	DOWN	H
**HALLMARK apoptosis**	130	0.017	DOWN	H
**HALLMARK KRAS-signaling DN**	166	0.032	DOWN	H
**HALLMARK allograft rejection**	160	0.036	DOWN	H
**HALLMARK TNFA-signaling via NFKB**	163	0.040	DOWN	H
**Cell Differentiation Process**				
**Boquest stem cell DN**	186	0.05	DOWN	CGP
**Sarrio epithelial mesenchymal transition UP**	149	0.006	DOWN	CGP
**Le neuronal differentiation DN**	16	0.034	DOWN	CGP
**P53-Mediated Cell Cycle Arrest and Cellular Senescence Process**				
**Wu apoptosis by CDKN1A via TP53**	47	0.002	DOWN	CGP
**Tang senescence TP53 targets DN**	48	0.00	DOWN	CGP

**a.** Official name of the biological process. **b.** Number of genes in the biological process. **c.** The FDR *q*-value estimates the significance of the enrichment of a biological process or a pathway. FDR *q*-values <= 0.05 are considered acceptable. **d.** Type of regulation of the genes involved in a process or a pathway. **e.** Name of the ontology defining a biological process or pathway. GO BP: gene ontology biological process; H: hallmark pathways; CGP: chemical and genetic perturbations pathways.

## Data Availability

The gene expression profiles of the patients and healthy controls are accessible in the Gene Expression Omnibus repository (GEO accession: GSE204775).

## Supplementary Materials

The following supporting information can be downloaded at https://www.mdpi.com/article/10.3390/life12070988/s1, Figure S1: Quality control evaluation sample 19; Figure S2: Enrichment plots of representative over represented biological processes in SoS fibroblasts samples by GSEA; Figure S3: Enrichment plots of representative down represented of C2,CGP and hallmark gene sets in SoS fibroblasts samples by GSEA; Table S1: Sotos patients enrolled; Table S2: Healthy parents enrolled; Table S3: Primers used for validation of microarray results by real-time PCR; Table S4: GSEA analysis.
