# Supplementary material for: NSD1 Mutations in Sotos Syndrome Induce Differential Expression of Long Noncoding RNAs, miR646 and Genes Controlling the G2/M Checkpoint
Source: Life (Basel). 2022 Jul 2;12(7):988. doi: 10.3390/life12070988 (PMC9324496; doi:10.3390/life12070988)
Supplement: Supplementary file 1 [file life-12-00988-s001.zip › life-1766636-supplementary.pdf]

Supplementary Tables

TableS1. Characteristics of healthy parents			
**Number**	**Sex[a]**	**Nucleotide change**	**Predicted protein change**
1	M	c.4497+1_4497+7delinsTCTGGA	p.?
2	M	del ex6	p.?
3	F	c.1633delA	Thr545Argfs*10
4	M	c.2386_2389del	Glu796Ilefs*10
5	M	c.6259-2A>G	p.?
6	F	c.6485A>G	His2162Arg
7	M	c.5644_5655delinsTGTA	Val1882Cysfs*7
8	F	del5q35.2-q35.3	
9	F	c.5146+1G>A	p.?
10	M	c.6010-10G<A	p.?
11	F	c.3958C>T	Arg1320*
12	M	c.6047_6050dup	Phe2018Serfs*12
13	M	del5q35.2-q35.3	
14	M	del5q35.2-q35.3	
15	M	c.1482C>A	Cys494*

[a] M: Male; F: Female

Table S2. Characteristics of healthy parents		
**Number**	**Sex[a]**	**Identifier in our previous study**
1	F	Ctr443
2	F	Ctr848
3	M	Ctr626
4	M	Ctr840
5	M	Ctr002

[a] M: Male; F: Female

Table S3. Primers of differentially expressed genes used for validation by real-time PCR

Gene Symbol	RefSeq Tran-script ID	Forward Primers	Reverse Primers	Product size (bp)	Temp
NSO3	NM_000603	5’CCAGCTAGCCAAAGTCACCAT3’	5’GTCTCGGAGCCATACAGGATT3’	175	60°C
CD19	NM_001770	5′TCAGCTGTGACTTTGGCTTATCTG3′	5’AGTCATTCGCTTTCTTTTCCT3’	120	60°C
NDRG2	NM_001282213	5’ACTGTCTATGGCACCCCCAA3’	5’TCGAACTGAAACAGTGGCTGG3’	188	60°C
SFN	NM_006142	5’CAGGCTACTTCTCCCCTCCT3’	5’TCAATCTCGGTCTTGCACTG3’	100	60°C
SORBS1	NM_001034954	5’CAGGCTACTTCTCCCCTCCT3’	5’TCAATCTCGGTCTTGCACTG3’	119	60°C
lnc-C00665	NC_000019.10	5’GTTTCACCATGTTGGCCAGG3’	5’GAAAACTCTGCCAGGGAGCA 3’	45	60°C
lnc-C2orf84-1	NM_001040710	5’GCCAAGAACACACTCTCATT3’	5’GTGGTCTGAAGCATCTGATTC3’	50	60°C
lnc-C20orf197	NM_173644	5’TGCTGGATACCTGAGGACTC3’	5’CTGGTCTAAGGTATCTTTGC3’	20	60°C
MIR646	NR_046099	5’GTGACAGATCTGAGACAAAC3’	5’CAAGCTGGTCCCTTTAATGG3’	22	60°C
ZNF883	NM_001101338	5’TGACCGCGAACCCATATCTC3’	5’GTTTCTCTCCAGTATGTATTC3’	28	60°C
GSC	NM_173849	5’GTTCAGCATCGACAACATCC3’	5’TAGAAGGCGCCATAGTCCGA3’	150	60°C
lnc-C15orf57	NM_052849	5’ATGTTTGAGAGCGCTGACTC3’	5’TCAGGGCAAGAATCCACAAA3’		60°C

Table S4. Significantly enriched gene sets in SoS samples by GSEA

Pathway or Process Description [a]	Number of Genes[b]	FDR q-Value[c]	Type of Regulation[d]	Ontology[f]
**Cancer process**				
Florio neocortex basal radial glia DN	151	0.00	DOWN	CGP
Rosty cervical cancer proliferation cluster	114	0.00	DOWN	CGP
Kong E2F3 targets	77	0.00	DOWN	CGP
Lee early T lymphocyte up	85	0.00	DOWN	CGP
Kobayashi EGFR signaling 24hr DN	213	0.00	DOWN	CGP
Whitfield cell cycle literature	39	0.00	DOWN	CGP
Whiteford pediatric cancer markers	102	0.00	DOWN	CGP
Nakayama soft tissue tumors PCA2 UP	76	0.00	DOWN	CGP
Zhan multiple myeloma pr UP	35	0.00	DOWN	CGP
Pyeon HPV positive tumors UP	79	0.00	DOWN	CGP
Sotiriou breast cancer grade 1 VS 3 UP	127	3.65×10^{-4}	DOWN	CGP
Chiang liver cancer subclass proliferation UP	146	8.15×10^{-4}	DOWN	CGP
Farmer breast cancer cluster 2	29	0.001	DOWN	CGP
Villanueva liver cancer KRT19 UP	147	0.002	DOWN	CGP
Rodrigues thyroid carcinoma DN	66	0.002	DOWN	CGP
Finetti breast cancer kinome red	15	0.002	DOWN	CGP
Riggi Ewing sarcoma progenitor DN	158	0.002	DOWN	CGP
Azare neoplastic transformation by STAT3 DN	16	0.002	DOWN	CGP
West adrenocortical tumor markers UP	103	0.005	DOWN	CGP
Li Wilms tumor anaplastic UP	15	0.010	DOWN	CGP
Li prostate cancer epigenetic	27	0.014	DOWN	CGP
GAL leukemic stem cell DN	188	0.014	DOWN	CGP
Rickman head and neck cancer B	37	0.018	DOWN	CGP
Poola invasive breast cancer UP	228	0.019	DOWN	CGP
Sengupta nasopharyngeal carcinoma UP	240	0.024	DOWN	CGP
SMID breast cancer luminal A UP	68	0.025	DOWN	CGP
Vantveer breast cancer BRCA1 UP	28	0.027	DOWN	CGP
Winnepenninckx melanoma metastasis UP	134	0.029	DOWN	CGP
Ferreira Ewings sarcoma unstable VS stable UP	131	0.029	DOWN	CGP
Lopes methylated in colon cancer UP	22	0.047	DOWN	CGP

Chiaradonna neoplastic transformation KRAS UP	113	0.047	DOWN	CGP
Andersen cholangiocarcinoma class2	143	0.048	DOWN	CGP
Boyault liver cancer subclass G5 DN	20	0.048	DOWN	CGP
**Cell cycle and proliferation process**				
Boyault liver cancer subclass G23 UP	44	0.025	DOWN	CGP
Whitfield cell cycle literature	116	0.00	DOWN	CGP
Fischer G2 M cell cycle	191	0.00	DOWN	CGP
Croonquist NRAS signaling DN	55	0.00	DOWN	CGP
Molenaar targets of CCND1 and CDK4 DN	48	0.00	DOWN	CGP
Ishida E2F targets	39	0.00	DOWN	CGP
Reichert mitosis LIN9 targets	21	0.00	DOWN	CGP
Graham normal quiescent VS normal dividing DN	74	1.15×10^{-4}	DOWN	CGP
Odonnell TFRC targets DN	110	2.18×10^{-4}	DOWN	CGP
Burton adipogenesis 3	85	3.49×10^{-4}	DOWN	CGP
Chemnitz response to prostaglandin E2 UP	112	0.001	DOWN	CGP
Graham cml dividing VS normal quiescent UP	157	0.002	DOWN	CGP
Osada ASCL1 targets UP	33	0.002	DOWN	CGP
Eguchi cell cycle RB1 targets	17	0.002	DOWN	CGP
Burton adipogenesis peak at 24HR	41	0.002	DOWN	CGP
Yu MYC targets UP	40	0.003	DOWN	CGP
Smirnov response to IR 6HR DN	88	0.003	DOWN	CGP
Zhou cell cycle genes in IR response 24HR	103	0.003	DOWN	CGP
Odonnell targets of MYC and TFRC DN	36	0.006	DOWN	CGP
Alcalay AML by NPM1 localization DN	158	0.010	DOWN	CGP
Zhou cell cycle genes in IR Response 6HR	70	0.011	DOWN	CGP
Hernandez mitotic arrest by docetaxel 1 UP	27	0.012	DOWN	CGP
Berenjeno transformed by RHOA forever DN	25	0.015	DOWN	CGP
Plasari TGFB1 targets 10HR DN	205	0.014	DOWN	CGP
Wilcox response to progesterone UP	125	0.018	DOWN	CGP
Chicas RB1 targets growing	196	0.02	DOWN	CGP

ULE splicing via NOVA2	35	0.02	DOWN	CGP
Benporath proliferation	124	0.033	DOWN	CGP
Whitfield cell cycle G2 M	162	0.034	DOWN	CGP
Liang silenced by methylation 2	50	0.034	DOWN	CGP
Nojima SFRP2 targets DN	19	0.034	DOWN	CGP
Kamminga EZH2 targets	37	0.037	DOWN	CGP
Graham CML quiescent_VS normal quiescent UP	74	0.040	DOWN	CGP
GOBP heat generation	15	0.014	UP	GO BP
GOBP regulation of meiotic cell cycle	31	0.07	UP	GO BP
GOBP negative regulation of nuclear division	42	0.016	UP	GO BP
GOBP regulation of feeding behavior	20	0.021	UP	GO BP
GOBP positive regulation of organic acid transport	29	0.017	UP	GO BP
GOBP regulation of nuclear division organization	106	0.046	UP	GO BP
GOBP kinetochore organization	16	0.041	UP	GO BP
HALLMARK G2M checkpoint	172	0.001	DOWN	H
HALLMARK KRAS signaling UP	174	0.0001	DOWN	H
HALLMARK mitotic spindle	163	0.008	DOWN	H
HALLMARK inflammatory response	159	0.006	DOWN	H
HALLMARK E2F targets	167	0.005	DOWN	H
HALLMARK apoptosis	130	0.017	DOWN	H
HALLMARK KRAS signaling DN	166	0.032	DOWN	H
HALLMARK allograft rejection	160	0.036	DOWN	H
HALLMARK TNFA signaling via NFKB	163	0.040	DOWN	H
**Cell differentiation process**				
Boquest stem cell DN	186	0.05	DOWN	CGP
Sarrio epithelial mesenchymal transition UP	149	0.006	DOWN	CGP
Le neuronal differentiation DN	16	0.034	DOWN	CGP
Mori immature B lymphocyte DN	74	0.02	DOWN	CGP
Hoffmann large to small pre bii lymphocyte UP	127	0.007	DOWN	CGP
**P53 mediated Cell cycle arrest and cellular senescence process**				
Wu apoptosis by CDKN1A via TP53	47	0.002	DOWN	CGP
Tang senescence TP53 targets DN	48	0.00	DOWN	CGP

a. Official name of biological process. **b.** Number of genes of biological process **c.** FDR q-value estimates the significance of the enrichment of a biological

process or a pathway. FDR q-value <= 0.05 are considered acceptable. **d.** Type of regulation of the genes involved in a process or a pathway. **e.** Name of the ontology defining a biological process or pathway. GO BP: stands for gene ontology biological process, H: Hallmark pathways, CGP: chemical and genetic perturbations pathways

Supplementary figures

Agilent SpikeIns Signal Statistics

Probe Name	Log(Relative Conc.)	Median(Log Proc. Sig.)	% CV	StdDev
(+)E1A_r60_3	0.30	0.38	54.90	0.24
(+)E1A_r60_a104	1.30	0.42	48.50	0.23
(+)E1A_r60_a107	2.30	1.45	37.38	0.17
(+)E1A_r60_a135	3.30	2.35	45.80	0.20
(+)E1A_r60_a20	3.83	2.54	39.87	0.15
(+)E1A_r60_a22	4.30	2.99	30.74	0.12
(+)E1A_r60_a97	4.82	3.76	51.01	0.23
(+)E1A_r60_n11	5.30	4.31	31.33	0.14
(+)E1A_r60_n9	5.82	4.31	35.96	0.17
(+)E1A_r60_1	6.30	4.83	41.69	0.16

Agilent SpikeIns: Log(Signal) vs. Log(Relative concentration) Plot

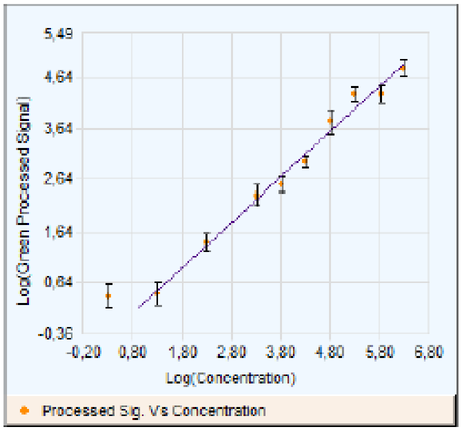

Agilent SpikeIns: %CV of Avg. Processed Signal Plot

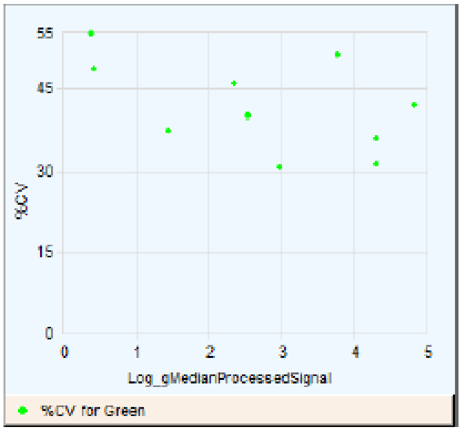

Median %CV:37.38

Agilent Spike-In Concentration-Response Statistics

Linear Range Statistics:

Low Signal	-0.14
High Signal	5.94
Low Relative Concentration	0.59
High Relative Concentration	7.47
Slope	0.88
R^2 Value	0.99

Signal Detection Limit Statistics

Saturation Point	5.89
Low Threshold	-0.25
Low Threshold Error	0.33
Spike-In Detection Limit	-0.62

Evaluation Metrics|for GE1_QCMT_Jun14
Good (5) ; Evaluate (5)

Metric Name	Value	Excellent	Good	Evaluate
IsGoodGrid	0.00		>1	<1
AnyColorPrcntFeatNonU...	0.06		<1	>1
gNegCtrlAveNetSig	17.88		<40	>40
gNegCtrlAveBGSubSig	-1.58		-10 to 5	<-10 or >5
gNegCtrlSDevBGSubSig	1.63		<10	>10
gSpatialDetrendRMSFil...	1.94		<15	>15
gNonCntrlMedCVProcSig...	39.64		0 to 8	<0 or >8
gE1aMedCVProcSignal	37.38		0 to 8	<0 or >8
absGE1E1aSlope	0.88		0.90 to 1.20	<0.90 or >1.20
DetectionLimit	-0.62		0.01 to 2	<0.01 or >2

• Excellent • Good • Evaluate

Supplementary Figure S1. Quality control report for sample 19. In table are reported in red parametric value that was not conformed to quality control, and in blu are indicated parametric value accepted by quality control.

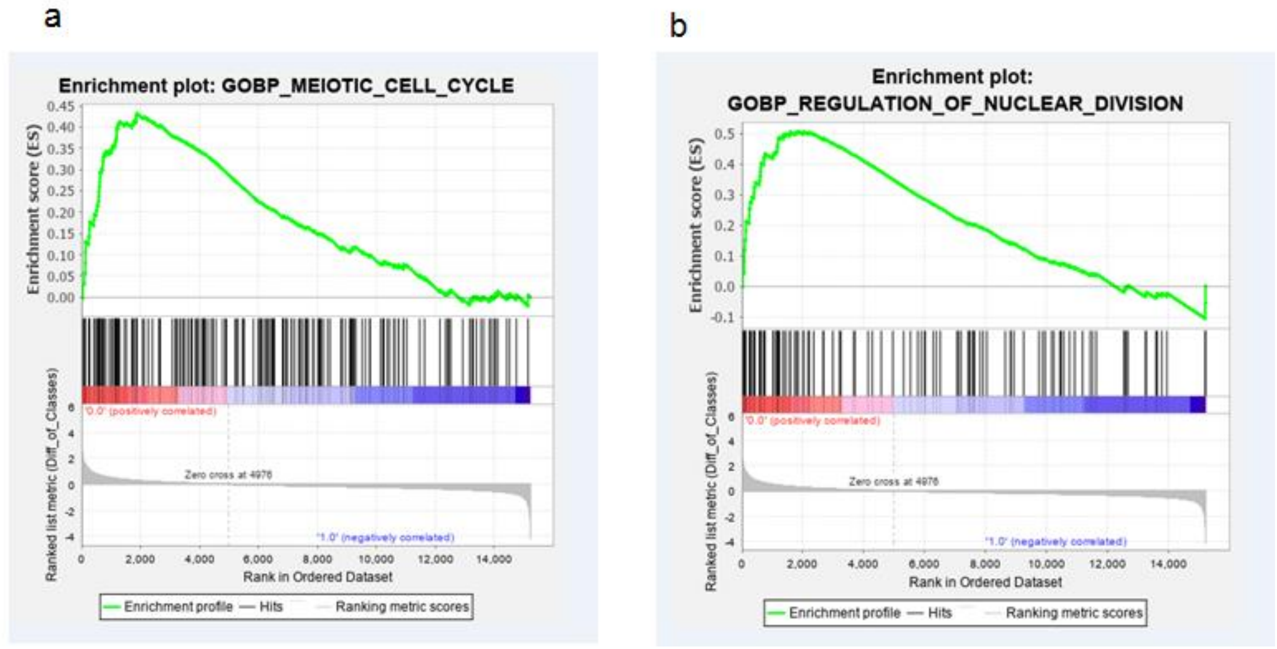

Supplementary Figure S2. Enrichment plots of representative over represented biological processes in SoS fibroblasts samples by GSEA. (a) Plots are relative to meiotic cell cycle **(b)** and regulation of nucleardivision.

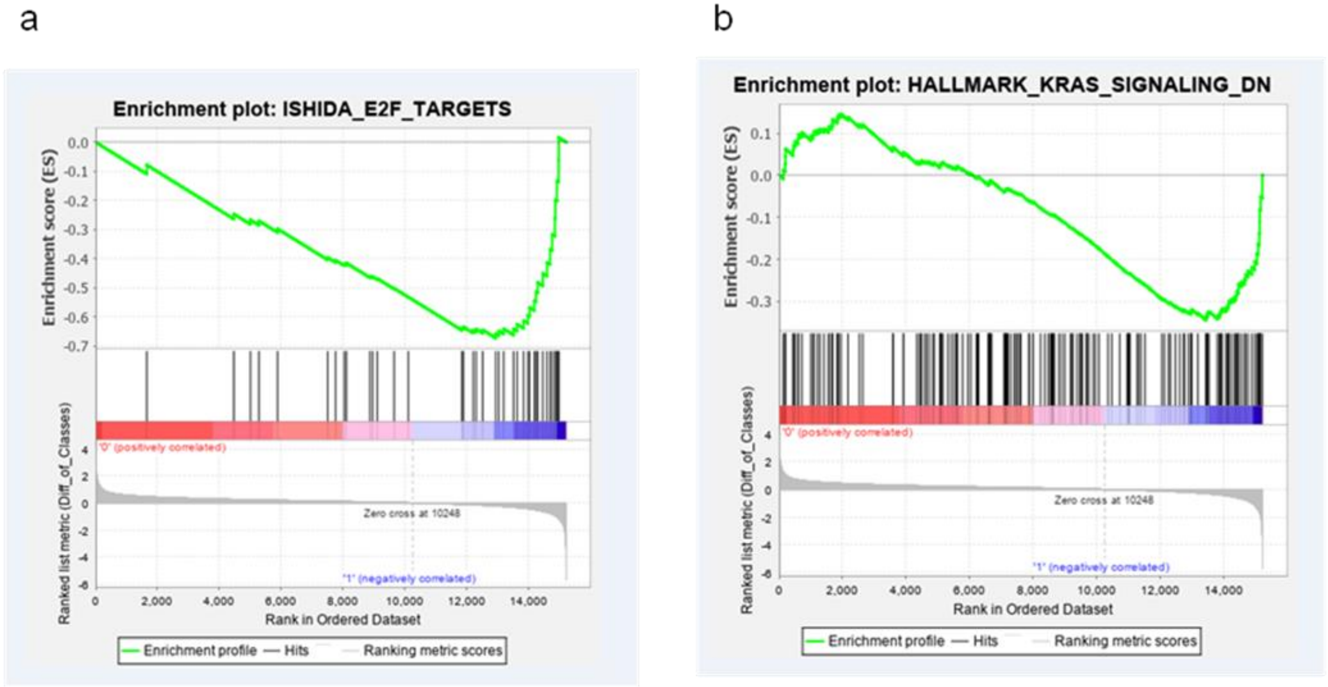

Supplementary Figure S3. Enrichment plots of representative down represented of C2,CGP and hallmark gene sets in SoS fibroblasts samples by GSEA. (a) Plots are relative to: E2F target **(b)** and KRAS signaling.
